# CAMP: Closed-Loop Acoustic-Magnetic Propulsion of Microrobots for Precision Cell Manipulation

**DOI:** 10.1002/aisy.202500300

**Published:** 2025-07-09

**Authors:** Fatma Ceren Kirmizitas, David P. Rivas, Max Sokolich, Jeffrey M. McNeill, Aditya Dutta, Sambeeta Das

**Affiliations:** Departments of Mechanical Engineering and Animal & Food Sciences, University of Delaware, Newark, DE 19716, USA; Department of Mechanical Engineering, University of Delaware, Newark, DE 19716, USA; Department of Mechanical Engineering, University of Delaware, Newark, DE 19716, USA; Department of Chemistry, University of Pennsylvania, Philadelphia, PA 19104, USA; Departments of Animal & Food Sciences, Biological Sciences, and Medical & Molecular Sciences, University of Delaware, Newark, DE 19716, USA; Department of Mechanical Engineering, University of Delaware, Newark, DE 19716, USA

**Keywords:** acoustic microrobots, cellular targeting, closed-loop control

## Abstract

Developing microrobotic systems for accurate and fast manipulation of microobjects or living cells has the potential to significantly advance biomedical and microfabrication applications. Despite recent progress in this field, comprehensive multistimuli responsive, fast, and precisely controllable microrobots remain limited. In this study, automated position and speed control of acoustically powered, bubble-based, magnetically steerable microrobots is demonstrated, along with micromanipulation of mammalian cells using these microswimmers. Enhanced control of the microswimmers is achieved by designing and implementing a closed-loop control system that guides the microrobots along a predetermined path while modulating their speed by adjusting the acoustic frequency near the resonant value. The microrobots are guided to cells, enabling cell manipulation by pulling them with the microrobots. Overall, the results highlight the capability and controllability of these magnetically and acoustically responsive microrobots for future cell-based applications, including manipulation, delivery, and microsurgery.

## 1. Introduction

In recent years, the potential of using microrobots for applications in cross-disciplinary fields such as robotics and control engineering, biomedical engineering, and chemistry has gained much attention. Owing to their untethered, controllable motion, small size, minimal invasiveness, and capacity to navigate into hard-to-reach areas, microrobots have been used in clot removal,^[1,2]^ single-cell manipulation,^[3,4]^ microsurgery,^[5,6]^ and drug delivery.^[7,8]^ Cell manipulation has become one of the most prominent techniques in the biomedical field, enabling researchers to study single-cell behavior and cell–cell communication. Traditionally, this task has relied on micromanipulators, which have required complex setups and skilled operators for the past decades. Common tools for single-cell manipulation include micropipettes,^[9]^ dielectrophoretic traps,^[10]^ optical tweezers,^[11]^ and acoustofluidic devices.^[12]^ Although effective, these traditional techniques have some disadvantages, such as operational complexity, low efficiency, and biocompatibility. In addition, microrobots offer an alternative approach for cell manipulation tasks at the intracellular and extracellular levels with high precision. Microrobots have several advantages over traditional approaches, including precise control, biocompatibility, lower costs, speed, and operational efficiency.^[13]^ A particularly notable advantage of microrobots is their untethered nature, which allows highly accurate, maneuverable, and controllable single-cell manipulation. Using microrobots, individual cells can be efficiently captured, transported, and precisely placed in designated locations. However, there are still several challenges to overcome in microrobotic-guided cell manipulation. To enable precise manipulation of cells and prevent misallocation of cells or cell damage, control and actuation mechanisms must be precise and viable in biological environments.

The actuation of microrobots can be achieved by external energy sources such as magnetic,^[8,14]^ acoustic,^[15,16]^ light,^[17,18]^ and electric fields;^[19,20]^ and also by utilizing energy sources in the medium, such as through chemicals;^[21,22]^ or employing living cells.^[23,24]^ These systems enable the actuation of mobile microrobots by harnessing environmental forms of energy, thereby eliminating direct contact with the power source. Nevertheless, it is crucial to take into account the biocompatibility and penetration capabilities of these actuation stimuli for further cell and tissue-based applications. For example, chemical-based actuation uses chemicals that are toxic to the cells, while light-based actuation relies on high-energy ultraviolet(UV) light which limits the penetration and can harm the cells irreversibly. In order to overcome this issue, external sources such as magnetic fields, acoustic waves, and their combination have emerged as promising actuation methods for navigating robots to accomplish specific tasks that include single-cell manipulation and targeted drug delivery. As both of these can be used for individual actuation systems, integrating magnetic field steering with acoustic actuation enhances the controllability of the robot’s motion and propulsion while maintaining biocompatibility. Acoustic waves have garnered interest in both microfluidics and biomedical applications due to their relatively low cost, biocompatibility, noninvasiveness, and ability to penetrate opaque tissues with low power. For example, Ahmed et al. have developed a neutrophil-inspired magnetically and acoustically responsive microrobot and demonstrated rolling motion along the endovascular walls.^[25]^ Moreover, red blood cells have been magnetically steered and acoustically driven using ultrasound in various biological fluids.^[26]^ Most recently, Aghahani et al. have developed a magnetically steered, microbubble-based, acoustically powered microrobot to show movement on different surfaces.^[15]^

Precise navigation is a crucial aspect of microrobot manipulation to effectively apply them across both in vitro and in vivo applications. One method to achieve robust control of microrobots is through a closed-loop control system, where precise feedback and control algorithms are combined to navigate the microrobot in an automated fashion.^[27]^ A closed-loop control system adjusts control parameters based on real-time feedback acquired from the microrobot’s movement to minimize deviations from the desired path and direct it to the target site. Closed-loop control strategies have been used previously for several magnetic microrobot designs.^[28–30]^ Our group has shown closed-loop control over several different shapes of magnetically responsive microrobots, such as bubble-propelled,^[31]^ catalytic Janus,^[32]^ and helical-shaped^[31]^ microrobots. Although acoustically powered microrobots commonly utilize open-loop control for manipulation tasks,^[12,33]^ the lack of positional feedback and hindered integration of control signals among the robots can pose challenges.

Recent approaches for the manipulation or transportation of a microparticle or single cell by acoustic microrobots are mainly based on an open-loop control strategy.^[12,34–37]^ The lack of a feedback system limits the precise motion and validation of microrobots for biomedical applications. Despite the high speed and strong potential of acoustically powered microrobots, there is a lack of demonstrated closed-loop control of these micromotors. Precise speed control is particularly important to ensure safe and accurate interactions with biological tissues, where any deviation in speed or direction could affect targeting and therefore treatment efficacy. Acoustic pressure-driven microrobots have been controlled via a closed-loop system,^[12,38]^ similar to acoustic tweezers. However, acoustic bubble-propelled motors, which are driven by the oscillation of an enclosed bubble and are more biocompatible, have yet to be controlled in such an automated manner. In addition, as the acoustically driven microrobots allow for high speeds, precise control of both the speed as well as directionality is still lacking.

Herein, we demonstrate for the first time the closed-loop navigation of an acoustically driven, bubble-propelled microrobot that follows predefined trajectories and whose speed is automatically modulated by a computer-controlled acoustic frequency. We also show the potential of using these multistimuli responsive microrobots for cellular manipulation. We apply open-loop and closed-loop control of acoustically powered magnetically steerable cup-shaped microrobots to show their efficiency and controllability. An open-loop control system allows us to detach a single cell from the cell cluster as well as transport the single cell in cell culture media. The closed-loop control system directs the microrobots in a predetermined path to reach a final destination. The microrobots can pick up and transfer cells to a nearby position and sort a single cell from a cell cluster with high precision and directional control.

## 2. Results

### 2.1. Acoustic Actuation of the Microrobots

**Figure 1.**
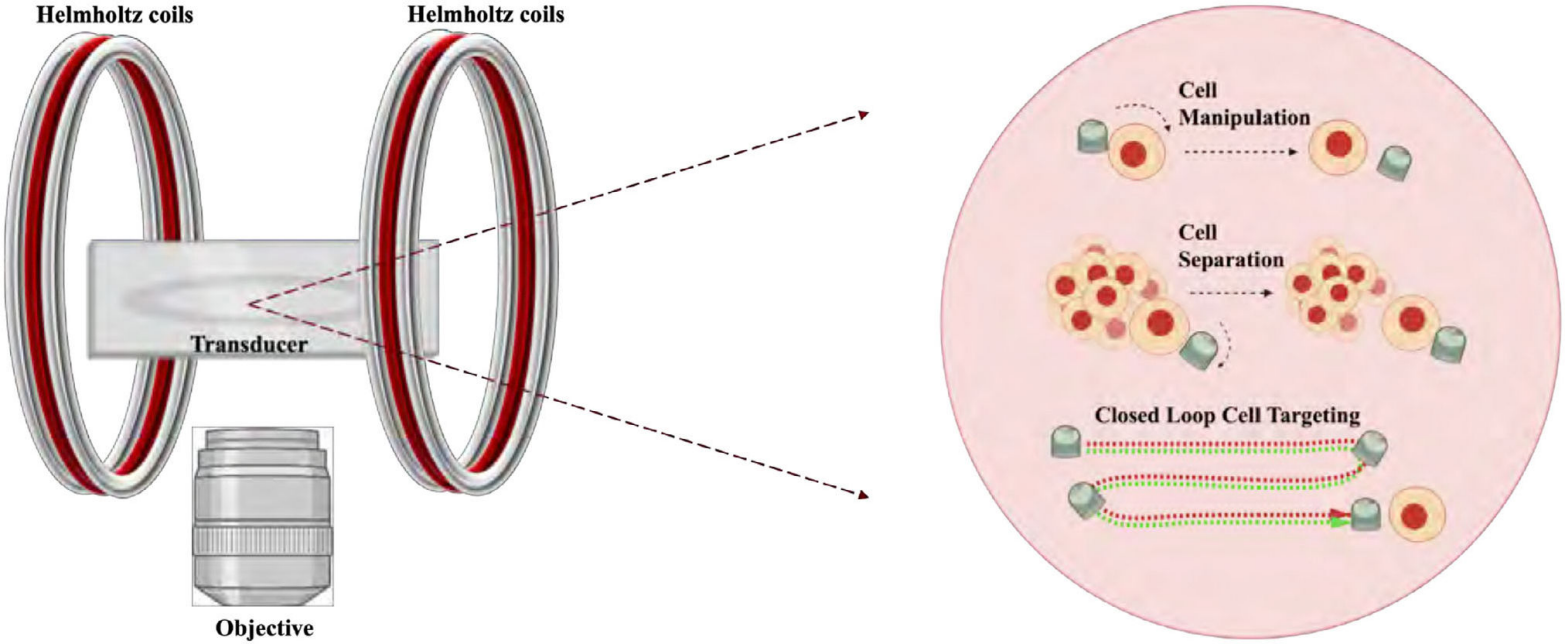
Schematic of the experimental setup (left) showing two of the six electromagnetic coils, the microscope objective, and the experimental workspace with a transducer to supply the acoustic waves. Shown on the right is a schematic of the acoustically powered microrobot carrying out cell manipulation tasks and following a drawn trajectory by computer-automated feedback control.

Figure 1 shows a schematic of the experimental setup and illustrates the manipulation tasks performed by the microrobots. The microrobots and cells were placed on a glass slide to which a transducer was attached to generate acoustic waves. Three pairs of electromagnets were used to provide magnetic fields that steered the microrobots.

The microrobots are actuated by the application of acoustic waves via a transducer. The acoustic waves cause a bubble that is trapped inside the microrobot to oscillate, which creates propulsive flows.^[15,34,39]^ There are two relevant forces produced by the acoustic waves: the secondary Bjerknes force and the streaming propulsive force. The secondary Bjerknes force acts to attract the oscillating microbubble (and hence the microrobot) toward the substrate, while the acoustic streaming force acts to propel the microrobot away from its open end. When the acoustic field is first applied, the microrobot tends to stand up with the open end facing the substrate. This is due to an imbalance of torques created by the attraction of the bubble to the substrate and the weight of the microrobot. Once it is in the upright position, the propulsive force due to acoustic streaming balances the downward force of attraction due to the secondary Bjerknes force. Tilting the microrobots by applying magnetic fields creates an asymmetry, breaking the equilibrium and resulting in a net force in the horizontal direction due to the acoustic streaming propulsive force. Therefore, tilting the microrobots in different directions enables directional control of their motion. The microrobot speed is determined by the amplitude of the acoustic waves and the difference between the frequency of the waves and the resonant frequency of the microbubble. An increase in acoustic amplitude increases the strength of the propulsive force, while the frequency difference influences the efficiency of energy transfer to the bubble, impacting both the speed and stability of microrobots’ motion.

### 2.2. Cell Manipulation

**Figure 2.**
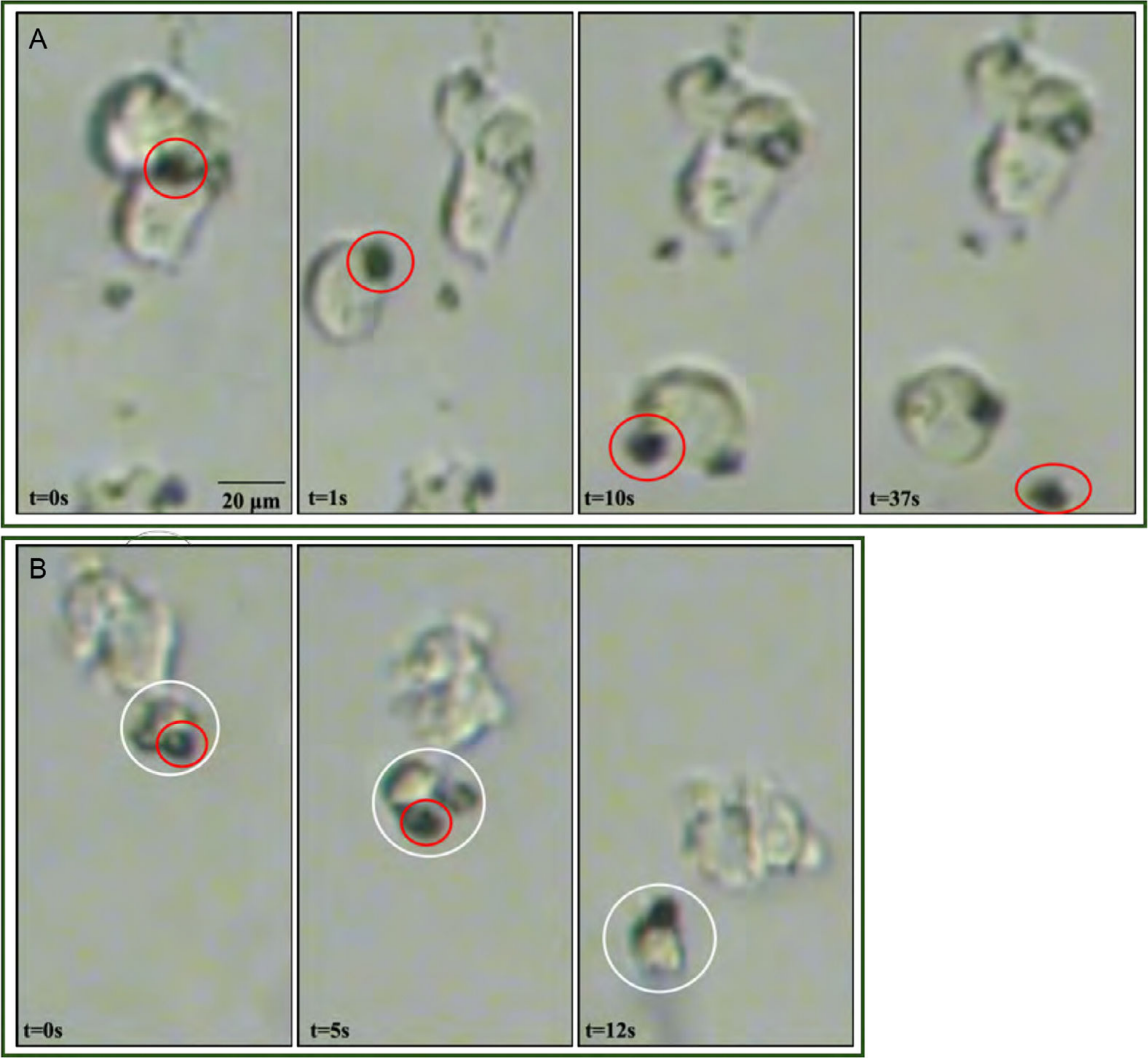
Cell manipulation and separation of CHO cells with a microrobot. A) Images of a microrobot picking and releasing a single CHO cell. B) Images of a CHO cell detachment from a cell cluster via a microrobot.

To demonstrate the efficiency of cell manipulation using acoustic microrobots, we used Chinese Hamster Ovary (CHO) cells (≈15 μm) to perform cell delivery experiments. Figure 2 and Video S1, Supporting Information, show a microrobot (circled in red in the figure) that approaches, picks up, and transports a free-standing CHO cell in the cell culture media to a new location. After transport, the microrobot can release the cell by pushing the cell away due to the fluid flow coming through its open end.

Another example is given in Figure 2, where two microrobots that were attached to the target CHO cell can rotate and detach the cell from the suspension cell cluster. Such capabilities could be used in cell separation and sorting and could have future relevance for microrobot-based microsurgery.

### 2.3. Closed-Loop Directional Control

**Figure 3.**
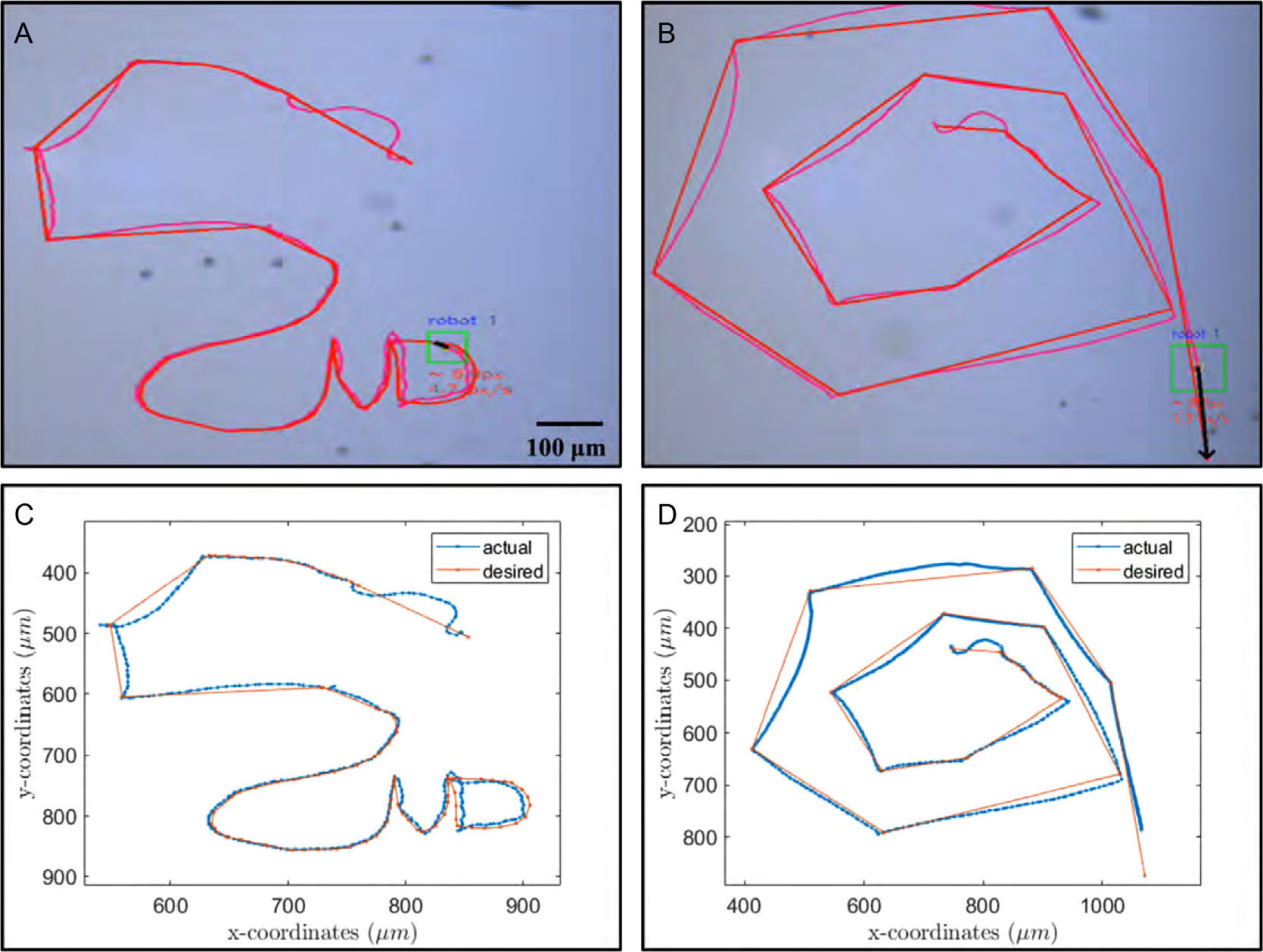
Two experimental trajectories of acoustic microrobots. Closed loop control of microrobot to follow the predefined A) “UD” and B) spiral loop trajectories and C–D) The comparison between the desired and actual trajectories of the microrobot, respectively. The curved trajectories are made up of many discrete nodes to which the microrobot is directed before targeting the next node of the path. Straight line segments correspond to cases in which the microrobot is directed to a single node at a distance rather than many nodes along a path.

Magnetic fields were applied using a custom-built electromagnetic system. The electromagnets were controlled via Python software which allowed for the use of both a game controller joystick control as well as automated computer control. Details of the electromagnetic system are provided in the experimental section. As shown in Figure 3 and Video S2, Supporting Information, the microrobot tracked the prescribed path (a spiral loop and the letters “UD”). The microrobot accomplished the user-defined trajectories with an average deviation between the desired and actual trajectories of 8.5 μm for the spiral loop and 6 μm for the “UD” trajectories.

In order to attain closed-loop control over the microrobot’s motion, we used an algorithm that adjusts the direction of the applied magnetic field to direct the microrobot to the desired target location.^[32]^ This is accomplished by evaluating the microrobot’s direction of motion and determining the offset between this and the applied magnetic field. This offset is used as a correction to the direction of the applied magnetic field to direct the microrobot toward the target location. For example, if the microrobot’s velocity points in the same direction as that of the magnetic field, the field is oriented along the vector that points from the microrobot toward the target location. Accounting for deviations by constant adjustments of the mapping between the field direction and the direction of motion of the microrobot is needed to assure robust performance in cases where the microrobot does not move in exactly the direction of the field or if there is a slight offset of the camera axis from that of the magnetic axis.

**Figure 4.**
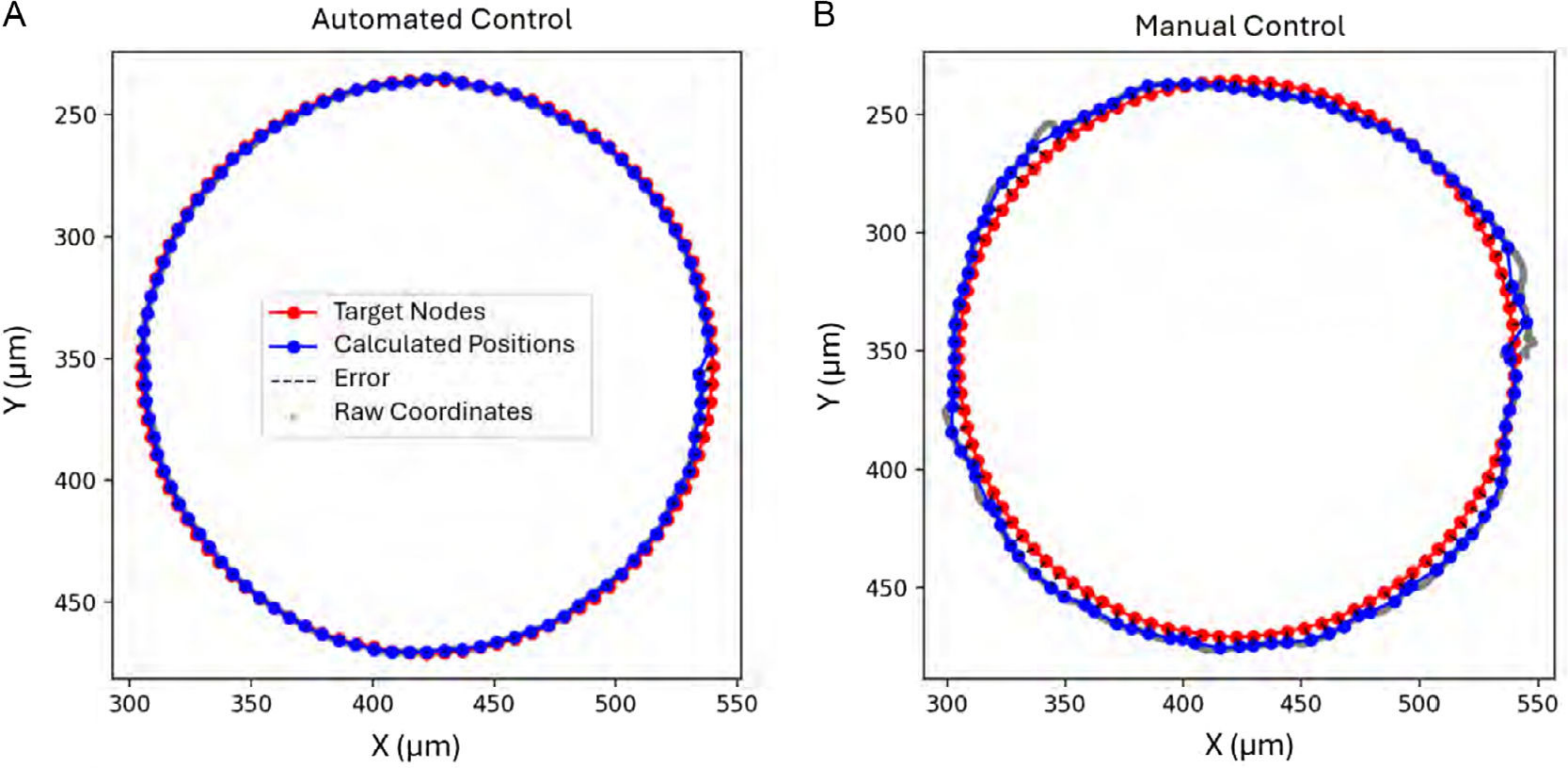
Comparison of A) automated and B) manual path following using closed-loop and open-loop control, respectively. The red points are the target nodes of the circular trajectory, while the blue points are the closest points on the microrobot trajectory to those target nodes. Black dashed lines represent the distance between these two, and gray points are the raw data of the microrobot coordinates in each frame. The average deviation between the target nodes and the actual trajectory for the closed-loop and open-loop cases was 1.1 μm and 4.1 μm, respectively.

In order to assess how accurately the microrobot follows a prescribed trajectory with both closed-loop and open-loop control, we guided a microrobot along a circular path using both automated and manual control and calculated the mean deviation between the desired and actual trajectories. The results are shown in Figure 4, and the corresponding video is provided in the SI (Video S6, Supporting Information). The accuracy was evaluated by taking the average distance between the target nodes and the closest point in the microrobot trajectory, resulting in an average deviation between the desired and actual path of 1.1 μm for the closed-loop case and 4.1 μm for the open-loop case. We note that segmenting the trajectory into multiple nodes rather than targeting a single node at a large distance resulted in increased accuracy, as can be seen in Figure 3 and Figure 4.

### 2.4. Automated Speed Control

**Figure 5.**
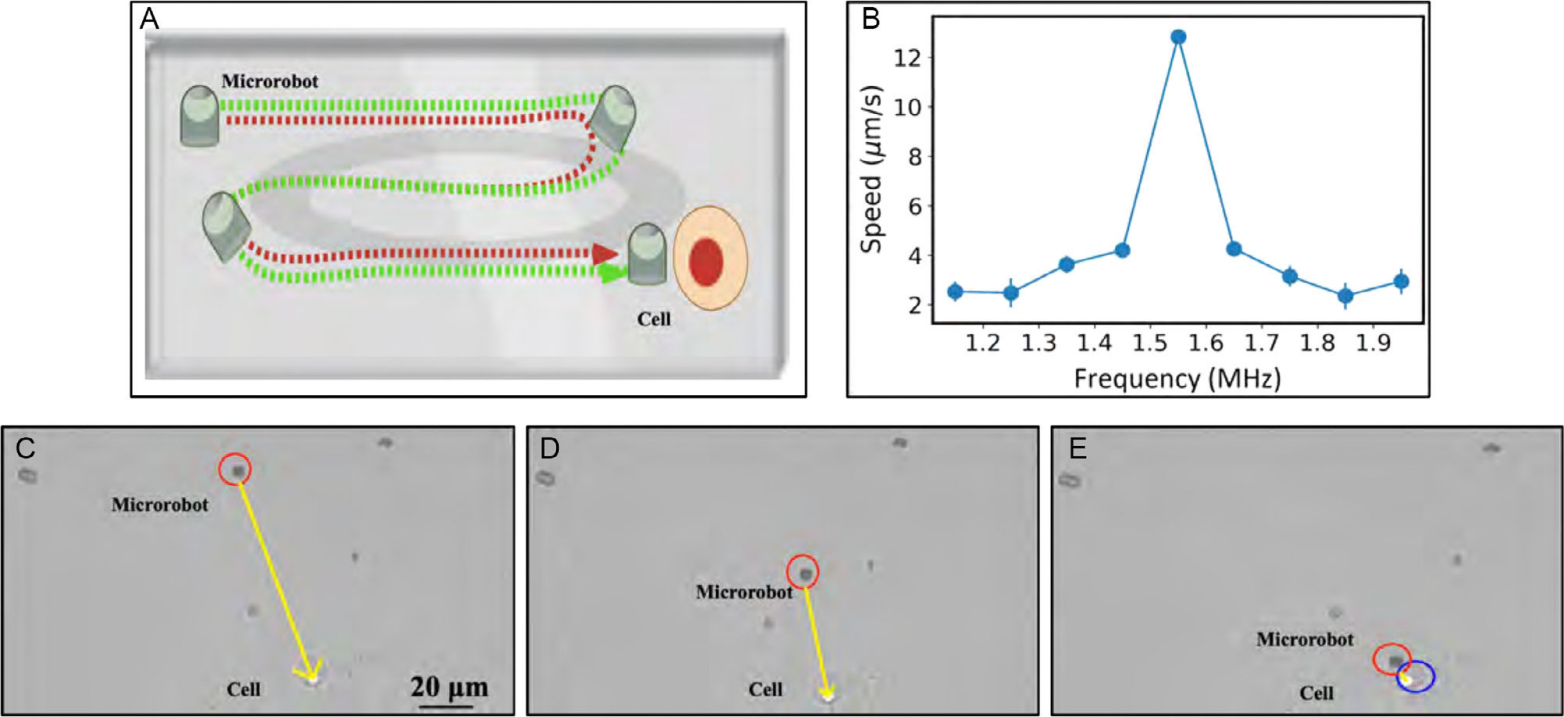
A) A schematic representation of the navigation of the microrobots to the target cell. B) Characterization of speed versus acoustic frequency for the microrobots under a constant magnetic field. C–E) The microrobot is steered to the cell under a constant magnetic and acoustic field by applying a closed-loop control system.

In order to control the speed of the microrobots, we also implemented an automated control of the acoustic frequency. An example of the speed of a microrobot as a function of acoustic frequency is shown in Figure 5B, which demonstrates the strong dependence of the speed as a function of frequency around the resonant value. An outline of the algorithm that controls the speed of the microrobot is given in Algorithm 1. First, a starting frequency of 1.2 MHz was applied, and the speed of the microrobot was determined. If the microrobot’s speed was below a threshold value, the frequency was increased by 0.1 MHz every 10 frames, which allowed for enough time to compute the average velocity of the microrobot at the new frequency. Once the microrobot reached a speed that was above the set threshold, the frequency was maintained. If the speed decreased again below the minimum threshold, the frequency was again increased until the maximum of 1600 kHz was reached, at which point the frequency was reset to the minimum frequency of 1.2 MHz and increased by 0.05 MHz increments. If the speed rose above a maximum threshold, the frequency was decreased by 75 kHz and the speed was reevaluated. If the speed was still too large, the frequency was again decreased; otherwise, the frequency was increased until the speed was above the minimum threshold. Video S3, Supporting Information, shows the automated speed control of a microrobot that followed a prescribed path. The frequency is shown in the upper left corner of the frames. The voltage (which controls the acoustic amplitude) is set to 1 volt. As can be seen in the video, an initial frequency of around 1.2 MHz was sufficient to actuate the microrobot at the desired speed. However, the speed decreased below the minimum threshold at around 56 s at which time the algorithm incrementally increased the frequency until stopping at ≈1.37 MHz when the microrobot had attained sufficient speed once again.

**Algorithm 1:** Acoustic Microrobot Automated Control Algorithm

**input**: Micro-robots Velocity Magnitude $\leftarrow v_{\mathrm{mag}}$
**output**: Acoustic Wave Generator Frequency $\leftarrow f_{\mathrm{current}}$
1 initialization;
2 let $f_{min}\leftarrow 1.2\;MHz$;
3 let $f_{max}\leftarrow 1.6\;MHz$;
4 let $f_{\mathrm{current}\;}\leftarrow f\;min$;
5 let $v_{min}\leftarrow 2\mu m/s$;
6 let $v_{max}\leftarrow 5\mu m/s$;
7 let increment←0.1MHz;
8 let $f_{\mathrm{optimal}\;}\leftarrow None$;
9 let n←0;
10 **White** True do
11 if $v_{\mathrm{mag}}<v_{\min}$ **then**
12 if $f_{\mathrm{current}}<f_{\max}$ **then**
13 **if** *n*%10 == 0 **then**
14 $f_{\mathrm{current}}\leftarrow f_{\mathrm{current}}+increment$
15 **else if** $f_{\mathrm{current}}>f_{\max}$ **then**
16 increment ← increment/2
17 $f_{\mathrm{current}}\leftarrow f_{\min\;}$
18 **else if** $v_{\min\;}<v_{\mathrm{mag}}<v_{\max\;}$ **then**
19 $f_{\mathrm{optimal}\;}\leftarrow f_{\mathrm{current}}$
20 increment ← increment/10
21 **else if** $f_{\mathrm{current}}>f_{\min\;}$ **then**
22 **if** *n*%20 == 0 and $f_{\text{current}}\leftarrow f_{\text{min}\;}$ **then**
23 $f_{\mathrm{current}}\leftarrow f_{\mathrm{current}}-75\;kHz$
24 *n*+ = 1
25 **end**

We note that the response time of our system is mainly determined by the frame rate of the camera, which was ≈24 fps in our experiments. We found that this rate was sufficient for tracking and guiding the microrobot accurately in all of our experiments.

### 2.5. Navigation of the Microrobots to Cells

In this section, we aimed to show the potential of our microrobot to target a single cell in biological fluid via a closed-loop control system. The combination of efficiency, precise directionality, and independent propulsion makes bubble-based microrobots an ideal tool for the selective manipulation of objects, particularly mammalian cells. To demonstrate the steering of microrobots in mammalian cells, a 2% w/v solution of acoustic microrobots was prepared in DMEM/F-12. About 5μl of the microrobot solution along with 5μl of CHO cellular suspension was added to the chamber. As shown in Figure 5(A) and Video S4, Supporting Information, once the target cell was determined, a desired microrobot was selected and was automatically directed to the cell using our closed-loop system. In the figure, the left side represents a schematic of the selected robot navigating to the cell, and on the right side, the real navigation process from Video S1, Supporting Information, is depicted from (C) to (D). The microrobot could reach the cell with precise control by following the predefined path. As a result of the accurate control of the microrobots, this study can be integrated into cell biology applications such as single-cell manipulation, cell and drug delivery, or transfection.

### 2.6. Cell Cytotoxicity

**Figure 6.**
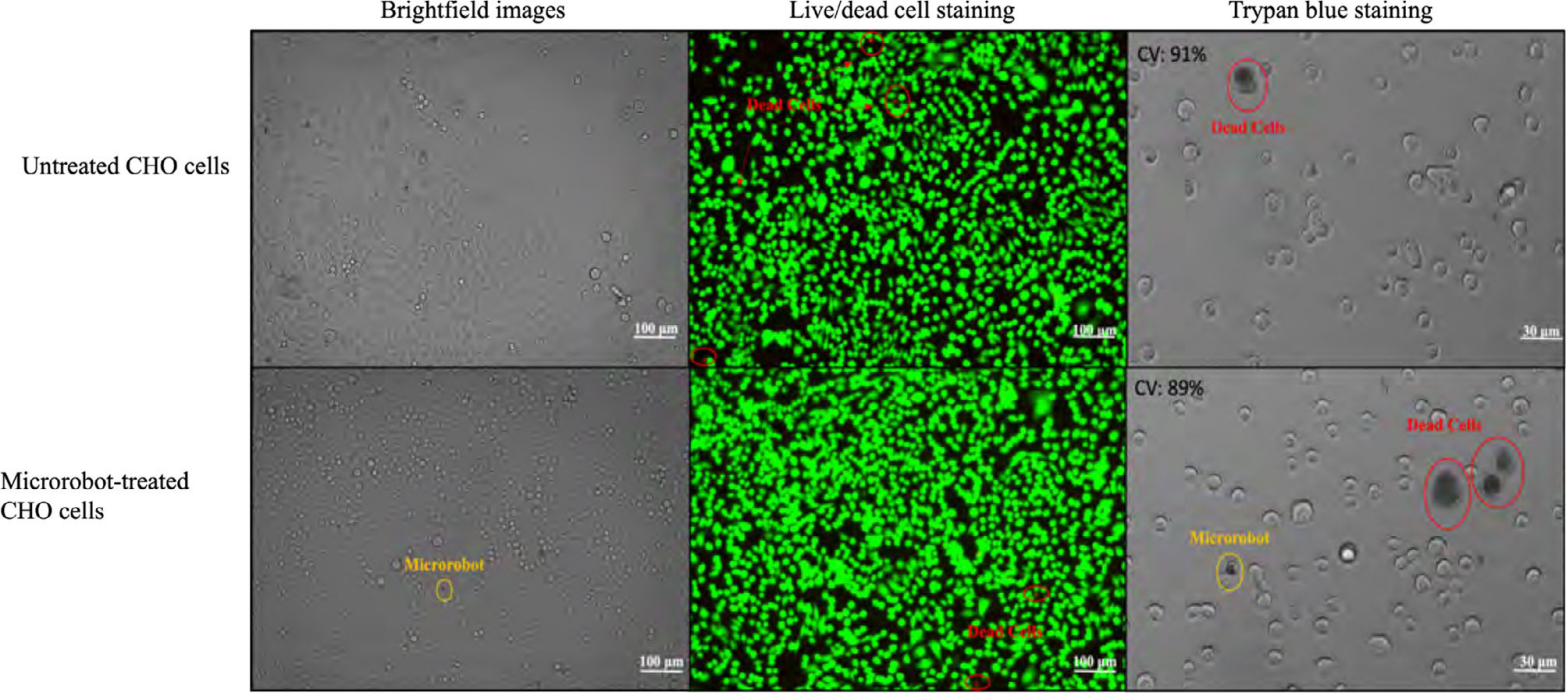
Images of CHO cells at 0 h and 24 h after microrobot actuation. Cell morphology was intact, and cell viability was 94% after treatment. Cells were stained with live (green) and dead (red) fluorescent dyes. Dead cells are shown in red circles. CV represents cell viability.

**Figure 7.**
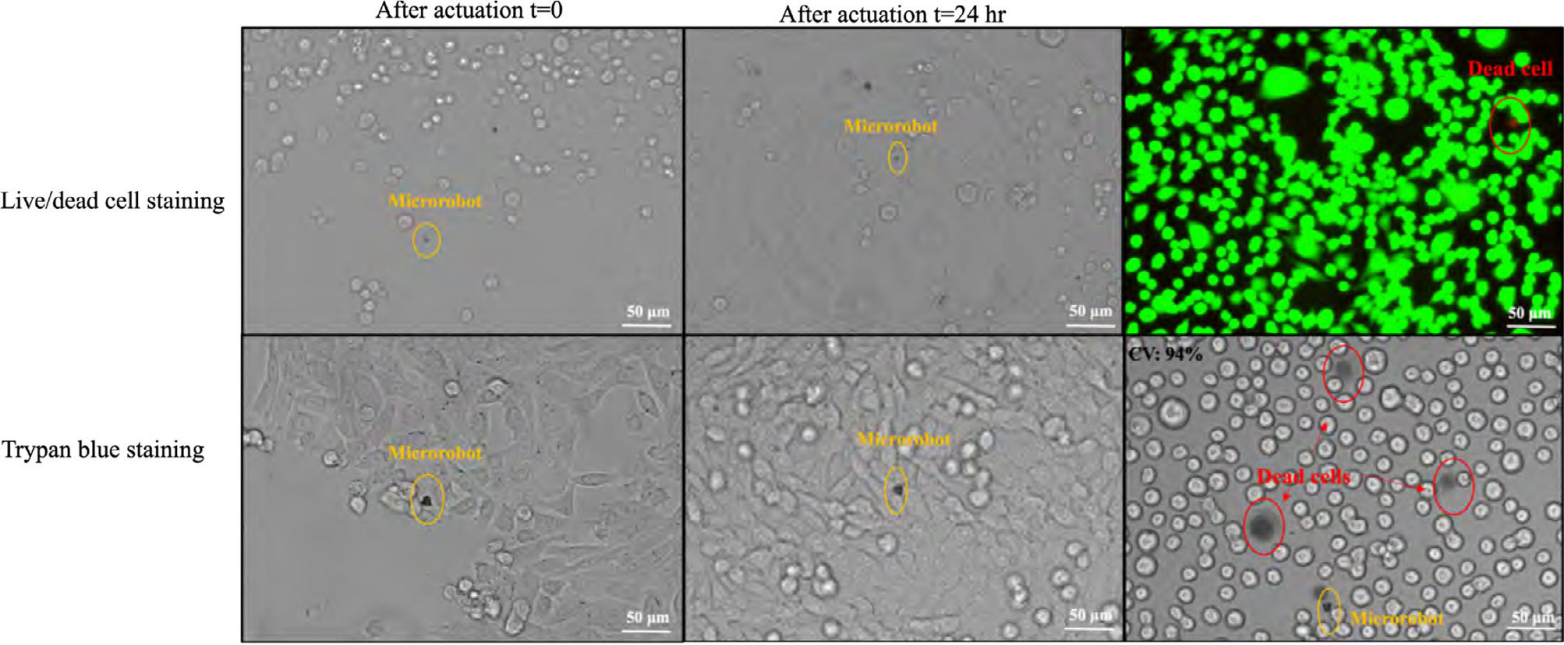
Microrobot-treated and untreated CHO cells after 24 h. Cell viability was observed via trypan blue staining assay and live/dead staining kit; dead cells are shown in red circles. Cells were stained with live (green) and dead (red) fluorescent dyes. CV represents cell viability.

For applications in cellular environments, especially with mammalian cells, microrobots must be biocompatible. To test this, CHO cells were selected to determine the biocompatibility of acoustic microrobots after 24 h of incubation and activation by trypan blue viability assay and live/dead viability/cytotoxicity kit for mammalian cells. Figure 6 shows images of cells immediately after and 24 h after microrobot treatment under constant acoustic frequency for 5 min. As can be seen in Figure 6, cells were viable, and cell morphology and proliferation were not affected after microrobot treatment compared to the control group. Trypan blue staining was performed to acquire quantitative cell viability for 24 h of incubation with microrobots. Figure 7 shows that the live/dead cell ratios for microrobot-treated and untreated cells were 89% and 91%, respectively. In addition, the cells that were in physical contact with the microrobot were still alive. The live/dead cell staining kit demonstrated the qualitative cytotoxic effect of microrobots on CHO cells after overnight incubation. The cell viability of untreated and treated cells was very similar, indicating that microrobots do not dramatically alter cell viability. The results revealed that the microrobots are well tolerated by the CHO cells and are likely to be nontoxic and biocompatible.

We note that we used a thin layer of nickel and a dilute concentration of microrobots, which limits toxic effects on cells; however, adding an additional layer of titanium or gold would further mitigate toxicity.^[40,41]^

## 3. Conclusion

In this work, acoustically powered, magnetically steerable, cup-shaped microrobots were successfully employed in simple yet rapid cell manipulation and cell targeting procedures with high biocompatibility. An open-loop control system enables us to move cells freely in the cell-dense environment and sort a single cell from a cell cluster. Employing a closed-loop control integration enabled additional precise control of microrobots’ trajectory and speed by magnetic steering and modulation of the acoustic frequency. In addition to the closed-loop path following via acoustic waves, we also were able to move the microrobots via closed-loop magnetic rolling (see Video S5, Supporting Information), offering an alternative method of control. This extends their capability by providing an additional means of reaching their target site, which could be beneficial in real-world applications where it may be difficult to apply a single method of actuation. Adapting the system for more demanding or complex tasks is feasible in several ways. For instance, the automated control system could be extended to operate in a serial manner, performing a sequence of drug delivery or cell manipulation tasks without human intervention. While global acoustic and magnetic fields apply uniform forces to all microrobots within a region, parallel operation could be achieved by using spatially partitioned fields, provided interference between them is minimal. This would require localized field generation, which introduces technical challenges, however. In more complex environments, obstacle avoidance may become necessary. To demonstrate this capability, we used our closed-loop system to guide a microrobot around passive spherical obstacles (Video S7, Supporting Information), illustrating the potential to operate effectively in cluttered or constrained environments.

Moreover, our results demonstrate the potential for bubble-driven microrobots to replace complex and expensive field-driven tweezing and actuation systems as a cost-effective and user-friendly alternative, thus broadening their capabilities.

## 4. Experimental Section

### Design and Fabrication:

**Figure 8.**
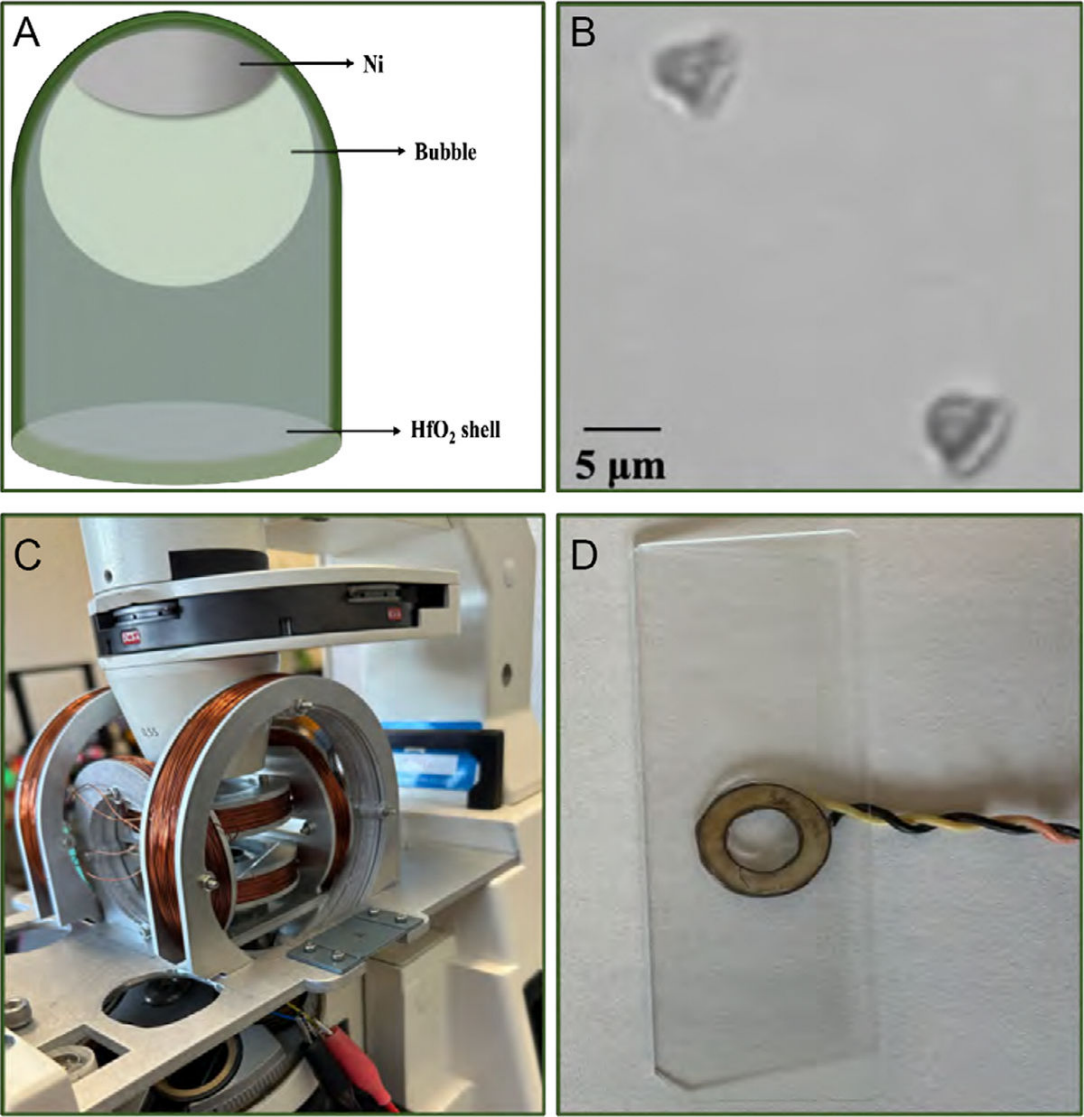
A–B) Schematic and optical microscopy image of cup-shaped microrobots. C) The 3D Helmholtz coil-based system mounted in the microscope. D) Representation of the acoustic slide prepared with the transducer.

The fabrication method of the bubble-driven acoustic microrobots used in this work was previously described by McNeill et al.^[39]^ Briefly, the microrobots were fabricated in large numbers using several evaporation and lithography steps. A bullet-shaped silica structure was fabricated by templating holes in a silicon wafer using shadow nanosphere lithography and creating a shell within those holes using area-selective atomic layer deposition. After freeing the shells with another etching step, a 10 nm layer of nickel was deposited on the capsules with electron beam deposition to make them magnetically responsive. Last, swimmers were modified to be hydrophobic by heating them with trichlorosilane vapor at 70 °C for 15 min. The swimmers used here were templated with 3-micron polystyrene to create 4-micron diameter swimmers. The final cup-shaped structure is presented in Figure 8. The final bubble-driven microrobot was a cup-like-shaped capsule with an inner cavity that was filled with an air bubble for acoustic propulsion, and a thin Ni layer that made the robot responsive to an external magnetic field.

### Electromagnetic System:

The magnetic fields were produced using a Helmholtz-based electromagnetic system composed of pairs of coils along the x and y axes. The details of the design and computer interfacing have been described previously.^[42]^ Briefly, the amount and direction of current going through the coils were modulated to set the field strength and direction, while a graphical user interface designed in Python allowed for real-time tracking of the microrobots and other controls. The coils were fixed within a 3D-printed stage that fits onto the microscope.

### Experimental Setup:

A piezoelectric transducer was fixed to the bottom of a glass microscope slide for the experimental setup. Since the space inside the transducer can be seen as a chamber, it was used as the environment for the microrobots to swim. The transducer glass was placed in an Axiovert 200 inverted microscope. To generate the acoustic sound field necessary to actuate an acoustic microrobot, a signal generator capable of 1–3 MHz was required. We chose the HiLetgo DDS AD9850 direct digital synthesizer module. This is because it was able to generate sine wave frequencies from 0 to 40 MHz and was straightforward to implement with physical computing hardware like an Arduino or Raspberry Pi. The AD9850 applied signals at a fixed 1 V amplitude, which was powerful enough to actuate acoustic microrobots but often too powerful and resulted in the microrobots moving too fast to be tracked effectively. As a result, we incorporated an automated modulation of the microrobot’s speed by implementing a closed-loop frequency control, as described earlier. It would also be possible to modulate the microrobot’s speed by adjusting the applied voltage to the acoustic transducer, hence changing the amplitude of the acoustic field. We found this to be somewhat more difficult ito implement and therefore utilized the frequency response of the microrobots instead.

### Cell Culture Studies:

CHO cells were kindly provided by our collaborator Dr. Ron Weiss (Massachusetts Institute of Technology, Massachusetts, USA). CHO cells were used as a representative cell line for demonstrating cellular manipulation. CHO cells were grown in Dulbecco’s Modified Essential Medium/Nutrient Mixture F-12 (DMEM/F-12, Gibco, BenchStable, USA) media containing 10% fetal bovine serum and 1% penicillin–streptomycin in a humidified cell culture incubator at 37 °C with 5% CO_2_. The cells were used for manipulation before they reached 90% confluency and after the third passage. Before experiments, cells were washed with Dulbecco’s Phosphate Buffered Saline (DPBS) (Gibco, BenchStable, USA) and trypsinized to detach cells from the culture flask. Cells were immediately transferred to the acoustic microrobot chamber by using a micropipette and mixed with the microrobots. All experiments were performed using these cells. Cell viability was assessed by trypan blue viability assay and live/dead viability/cytotoxicity kit (Invitrogen, L3224) that can directly identify live and dead cells in the population. Cells were cultured with microrobots, and viability was determined after 24 h. Cells were seeded in a 6-well plate (Costar, Corning, USA) at a concentration of 1 × 10^5^ cells/ml per well and incubated at the same culture conditions with the 2% w/v microrobot solution for 24 h of incubation. Since dead cells typically float in the media, supernatants were collected, centrifuged, and suspended in DPBS. A 10 μl of cell suspension was mixed with an equal volume of 0.4% trypan blue, and cells were counted in a cell counter (Nexcelom Cellometer Vision Trio Cell Profiler). The cells were then imaged under an optical microscope (ZOE Fluorescent Cell Imager, USA). Following the trypan blue viability assay, cell morphology was observed under the optical microscope. Live/dead cell staining was performed according to the manufacturer’s protocol. Briefly, cells were stained with 0.1% calcein AM (green) and 0.2% ethidium homodimer-1 (red) in phosphate-buffered Saline (PBS) buffer for 25 min at 37 °C. Green and red fluorescent stains represent the live and dead cells, respectively. The effect of microrobot actuation on cell viability was also determined on CHO cells by following the same protocols. After navigating the microrobots with an approximate frequency of 1 MHz for 5 min, the cells were incubated overnight in a humidified cell culture incubator at 37 °C with 5% CO_2_. Cell morphology was observed under the optical microscope directly after and 24 h after actuation.

## Supplementary Material

video 3

video 1

video 4

video 2

video 6

video 7

video 5

Supporting Information

Supporting Information is available from the Wiley Online Library or from the author.

## Data Availability

The data that support the findings of this study are available from the corresponding author upon reasonable request.

## References

[1] Xie M, Zhang W, Fan C, Wu C, Feng Q, Wu J, Li Y, Gao R, Li Z, Wang Q, , Adv.Mater 2020, 32, 2000366. 10.1002/adma.202000366 32430939

[2] Wang Q, Wang B, Yu J, Schweizer K, Nelson BJ, Zhang L, In 2020 IEEE international conference on robotics and automation (ICRA), IEEE 2020, pp. 10285–10291.

[3] Villa K, Krejčová L, Novotný F, Heger Z, Sofer Z, Pumera M, Adv. Funct. Mater. 2018, 28, 1804343.

[4] Peng X, Urso M, Ussia M, Pumera M, ACS nano 2022, 16, 57615. 10.1021/acsnano.1c11136 35451832

[5] Vyskocil J, Mayorga-Martinez CC, Jablonská E, Novotny F, Ruml T, Pumera M, ACS nano 2020, 14, 8247. 32544324 10.1021/acsnano.0c01705

[6] Huang C, Lai Z, Wu X, Xu T, Cyborg Bionic Syst. 2022, 2022, 0004. 36924475 10.34133/cbsystems.0004 PMC10010670

[7] Lee H, Kim D.-i., Kwon S.-h., Park S, ACS Appl. Mater. Interfaces 2021, 13, 19633. 33877809 10.1021/acsami.1c01742

[8] Alapan Y, Bozuyuk U, Erkoc P, Karacakol AC, Sitti M, Sci. robotics 2020, 5, eaba5726. 10.1126/scirobotics.aba5726 33022624

[9] Lu Z, Moraes C, Ye G, Simmons CA, Sun Y, Plos one 2010, 5, e13542. 21042403 10.1371/journal.pone.0013542 PMC2958835

[10] Voldman J, Annu. Rev. Biomed. Eng. 2006, 8, 425. 16834563 10.1146/annurev.bioeng.8.061505.095739

[11] Mirsaidov U, Scrimgeour J, Timp W, Beck K, Mir M, Matsudaira P, Timp G, Lab Chip 2008, 8, 2174. 19023484 10.1039/b807987k

[12] Dai Nguyen T, Fu YQ, Tran V-T, Gautam A, Pudasaini S, Du H, Sens. Actuators, B: Chemical 2020, 318, 128143.

[13] Chen W, Zhou H, Zhang B, Cao Q, Wang B, Ma X, Adv. Funct. Mater. 2022, 32, 2110625.

[14] Wang Q, Du X, Jin D, Zhang L, ACS nano 2022, 16, 604. 34985859 10.1021/acsnano.1c07830

[15] Aghakhani A, Yasa O, Wrede P, Sitti M, Proc. Natl. Acad. Sci. 2020, 117, 3469. 32015114 10.1073/pnas.1920099117 PMC7035478

[16] Del Campo Fonseca A, Glück C, Droux J, Ferry Y, Frei C, Wegener S, Weber B, El Amki M, Ahmed D, Nat. Commun. 2023, 14, 5889. 37735158 10.1038/s41467-023-41557-3 PMC10514062

[17] Kochergin YS, Villa K, Nemeskalova A, Kuchař M, Pumera M, ACS nano 2021, 15, 18458. 34730953 10.1021/acsnano.1c08136

[18] Huang J, Yu X, Li L, Wang W, Zhang H, Zhang Y, Zhu J, Ma J, ACS nano 2024, 18, 2006. 38205954 10.1021/acsnano.3c08277

[19] Alapan Y, Yigit B, Beker O, Demirörs AF, Sitti M, Nat. Mater. 2019, 18, 1244. 31235903 10.1038/s41563-019-0407-3

[20] Kwon GH, Choi YY, Park JY, Woo DH, Lee KB, Kim JH, Lee S-H, Lab Chip 2010, 10, 1604. 20376390 10.1039/b926443d

[21] Yang Y, Arqué X, Patiño T, Guillerm V, Blersch P-R, Pérez-Carvajal J, Imaz I, Maspoch D, Sánchez S, J. Am. Chem. Soc. 2020, 142, 20962. 33274916 10.1021/jacs.0c11061

[22] Feng Y, Yuan Y, Wan J, Yang C, Hao X, Gao Z, Luo M, Guan J, Appl. Phys. Rev. 2021, 8, 1.

[23] Dai Y, Jia L, Wang L, Sun H, Ji Y, Wang C, Song L, Liang S, Chen D, Feng Y, , Small 2022, 18, 2105414. 10.1002/smll.202105414 35233944

[24] Magdanz V, Khalil IS, Simmchen J, Furtado GP, Mohanty S, Gebauer J, Xu H, Klingner A, Aziz A, Medina-Sánchez M, , Sci. Adv, 2020, 6, 28 eaba5855. 32923590 10.1126/sciadv.aba5855 PMC7450605

[25] Ahmed D, Baasch T, Blondel N, Läubli N, Dual J, Nelson BJ, Nat. Commu. 2017, 8, 1. 10.1038/s41467-017-00845-5 PMC5626690 28974671

[26] Wu Z, Li T, Li J, Gao W, Xu T, Christianson C, Gao W, Galarnyk M, He Q, Zhang L, , Acs Nano 2014, 8, 12041. 25415461 10.1021/nn506200x PMC4386663

[27] Jiang J, Yang Z, Ferreira A, Zhang L, Adv. Int. Syst. 2022, 4, 2100279.

[28] Rahman MA, Takahashi N, Siliga KF, Ng NK, Wang Z, Ohta AT, Rob. Biomimetics 2017, 4, 1. 10.1186/s40638-017-0064-4 PMC5662703 29152448

[29] Khalil IS, Ferreira P, Eleutério R, de Korte CL, Misra S, In 2014 IEEE Inter. Conf. on Robotics and Automation (ICRA), IEEE 2014, pp. 3807–3812.

[30] Jiang J, Yang L, Zhang L, IEEE Robotics and Automation Letters 2021, 6, 827.

[31] Yang Y, Rivas D, Sokolich M, Das S, In 2023 Inter. Conf. on Manipulation, Automation and Robotics at Small Scales (MARSS)., IEEE 2023, pp. 1–6. 10.1109/marss58567.2023.10294166 PMC11221291 38962675

[32] Sokolich M, Rivas D, Shah ZH, Das S, MRS Adv. 2023, 1. 42281918 10.1557/s43580-023-00585-3 PMC13251878

[33] Cao HX, Nguyen VD, Jung D, Choi E, Kim C-S, Park J-O, Kang B, Pharmaceutics 2022, 14, 2143. 36297578 10.3390/pharmaceutics14102143 PMC9609374

[34] Ren L, Nama N, McNeill JM, Soto F, Yan Z, Liu W, Wang W, Wang J, Mallouk TE, Sci. Adv. 2019, 5, eaax3084. 31692692 10.1126/sciadv.aax3084 PMC6814402

[35] Ozcelik A, Rufo J, Guo F, Gu Y, Li P, Lata J, Huang TJ, Nat. Methods 2018, 15, 1021. 30478321 10.1038/s41592-018-0222-9 PMC6314293

[36] Ding X, Shi J, Lin S-CS, Yazdi S, Kiraly B, Huang TJ, Lab Chip 2012, 12, 2491. 22648600 10.1039/c2lc21021e PMC3991783

[37] Wu Y, Boymelgreen A, Yossifon G, Curr. Opin. Colloid Interface Sci. 2022, 101611.

[38] Wei Y, Lu X, Shen H, Peng H, Yuan Z, Guo X, Liu W, Adv. Mater. Technol. 2021, 6, 2100470.

[39] McNeill JM, Nama N, Braxton JM, Mallouk TE, ACS nano 2020, 14, 7520. 32432850 10.1021/acsnano.0c03311

[40] Wrede P, Degtyaruk O, Kalva SK, Deán-Ben XL, Bozuyuk U, Aghakhani A, Akolpoglu B, Sitti M, Razansky D, Sci. Adv. 2022, 8, eabm9132. 35544570 10.1126/sciadv.abm9132 PMC9094653

[41] Jeon S, Kim S, Ha S, Lee S, Kim E, Kim SY, Park SH, Jeon JH, Kim SW, Moon C, , Sci. Robotics 2019, 4, eaav4317. 10.1126/scirobotics.aav4317 33137727

[42] Sokolich M, Rivas D, Yang Y, Duey M, Das S, MethodsX 2023, 10, 102171. 37122368 10.1016/j.mex.2023.102171 PMC10133746

